# Immune Checkpoint Receptor/Ligand Expression and Chemotherapy in Colorectal Cancer

**DOI:** 10.3390/cancers15030914

**Published:** 2023-02-01

**Authors:** Roberto Benelli, Maria Raffaella Zocchi, Alessandro Poggi

**Affiliations:** 1 SSD Oncologia Molecolare e Angiogenesi, IRCCS Ospedale Policlinico San Martino, 16132 Genoa, Italy; 2 Division of Immunology, Transplantation and Infectious Diseases, Ospedale San Raffaele, 20162 Milan, Italy

## 1. Introduction

Immune checkpoint (IC) molecules act as receptors, expressed on immune effector cells, that are able to recognize specific ligands in normal or tumor cells. After the interaction of ICs and their ligands, immune cells downregulate effector functions, such as tumor cell cytolysis and the production of antitumor cytokines [1]. Eventually, immune cells become unresponsive, allowing tumor cell escape, proliferation, and spread. The use of antibodies specific to ICs, the so-called immune checkpoint inhibitors (ICI), can subvert tumor-mediated immune inhibition. This awakens effector cell response and leads to reductions in tumor mass [1]. Indeed, the use of ICI for the therapy of several kinds of tumors can greatly improve the clinical outcome in patients where chemo/radiotherapy are not effective [1]. Importantly, ICI is effective only when antitumor-specific T lymphocyte immunity can be triggered [1,2]. It is conceivable that treatment with ICI is optimal when these cells are already present at the tumor site and/or tumor-associated antigen-specific cytolytic T lymphocytes (CTL) can be elicited in regional lymph nodes next to primary and/or metastatic tumors [1,2]. Among the variety of IC molecules expressed by leukocytes infiltrating the tumor [1,2], cytotoxic T lymphocyte antigen-4 (CTLA-4) and programmed cell death receptor-1 (PD1) are the main targets of ICI therapy [1,2,3]. Besides the unequivocal antitumor effect of the anti-CTLA-4 and/or anti-PD1 antibodies, it is evident that not all patients can respond to this therapy [1]. Additionally, it is becoming clear that CTLA-4 and PD1 can also be expressed by tumor cells [4,5,6]. The effects of the interaction of ICI with these unconventional targets, besides effector T lymphocytes, are unclear. In T cells, ICIs block the negative signal delivered through the IC receptors, relieving the brake of antitumor immune response and triggering self-human histocompatibility antigen reactivity [3]. This is confirmed by the typical autoimmune side effects detectable after ICI therapy [1,2]. Another point of great interest is the impact of chemotherapy treatment on the expression of IC molecules and/or IC ligands on tumor cells [7]. In fact, ICI therapy is performed in combination with chemotherapy and there are several reports claiming that anti-proliferative drugs can affect the surface expression of IC molecules or their ligands [7].

## 2. Immune Checkpoint Receptors Expressed on CRC Cells. What Is Their Function? 

Derakhshani A. and colleagues have recently reported by bioinformatic analysis that CTLA-4 is overexpressed in tumor areas of colorectal carcinoma (CRC) compared to adjacent non-tumoral areas [8]. More interestingly, CTLA-4 expressed on some CRC cell lines, and in particular, on SW480 cells, is greatly downregulated by treatment with capecitabine. This drug is a 5-fluorouracil (5-FU)-related compound that can target the thymidylate synthase, leading to the blockage of DNA synthesis and eventually to the inhibition of tumor cell proliferation [9]. This finding is of great interest, as the role of CTLA- 4 on CRC cells is still undefined. Zhang H. and colleagues reported that the CTLA-4 expressed on tumor cells can play an active role in the fate of a subset of non-small cell lung cancer (NSCLC) [10]. Indeed, anti-CTLA-4 antibodies can induce PDL-1 expression through the activation of epidermal growth factor receptor (EGFR)-mediated signals, leading to MEK and ERK activation. Moreover, the anti-CTLA-4 antibody can also trigger the proliferation of NSCLC, both in vitro and in vivo [10]. The contribution of Derakhshani A. and colleagues indicates that CTLA-4 is also expressed in CRC and supports the rationale of testing the anti-CTLA-4 antibodies on CRC cell lines, to confirm the results reported for NSCLC.

According to these findings, it is possible to hypothesize that chemotherapy can influence CTLA-4 expression on CRC. 5-FU can dose-dependently upregulate the expression of PDL-1 on CRC or esophageal adenocarcinoma cell lines [11]. Notably, in this model, PDL-1 induction appeared to be independent of the well-known interferon (IFN)γ-mediated effect. Thus, chemotherapy with 5-FU-related compounds could doubly affect the clinical response to ICI therapy, both downregulating CTLA-4 pro-tumorigenic signaling and possibly increasing the PDL-1-mediated inhibition of specific T lymphocyte immune response. Altogether, these observations suggest the association of chemo- and ICI therapy with enhancing patients’ immune response. The exact schedule of administration for these two antitumor weapons is a new challenge, aimed to improve patients’ clinical outcomes. 

## 3. Chemotherapy and Immune Checkpoint Ligands. An Unexpected Link? 

In this context, the report from Kevin Chih-Yang Huang and colleagues is focused on the decitabine (DAC) effect on PDL-1 expression and function in CRC [12]. They demonstrated that DAC, inducing DNA hypomethylation through the inhibition of DNA methyltransferase 1 (DNMT1), leads to an increase in tumor PDL-1 expression. This effect was accompanied by a rise in immune-related gene expression within the tumor and the expansion of tumor T cell-infiltrating lymphocytes. The activity of DAC as a DNMT1 inhibitor needs DNA replication to show its effects. Accordingly, it works better on cells that divide quickly, such as tumor cells as compared to healthy cells. It is of note that DAC and oxaliplatin (OXP) can increase the immunogenicity of the tumor. This would suggest that chemotherapy can remodel the tumor microenvironment, favoring a stronger anticancer immune response, possibly leading to relevant clinical benefits to CRC patients in the near future.

Kevin Chih-Yang Huang and colleagues found that not only 5-FU but also OXP, irinotecan, or Doxorubicin can increase PDL-1 expression on different CRC cell lines. This effect was evident in a murine model using the murine cell line CT26 upon treatment with OXP or 5-FU [12]. More importantly, the increment in PDL-1 expression was also observed in tissue samples isolated from CRC patients treated with FOLFOX-based neoadjuvant chemotherapy compared to the tumor samples from the same patients before the treatment. Additionally, FOLFOX chemotherapy was associated with a strong increase in CD8^+^ tumor-infiltrating lymphocytes (TIL), which in turn produced IFNγ, leading to PDL-1 expression within the tumor. Different DNMT inhibitors such as 5′-azacitidine (5-AC) and SGI-1027 (SGI) also triggered PDL-1 upregulation in CRC cells, like the suppression of DNMT1 expression by silencing RNA. Conversely, the overexpression of DNMT1 had the opposite effect. The increased PDL-1 expression observed in CT26 CRC cells in vivo, upon DAC or OXP treatment, was accompanied by the upregulation of some cytokine-, chemokine-, and immune response-associated genes (TNF signaling, NF-kB activation, NOD-like receptor, and cytokine–cytokine receptor interaction) [12]. Altogether, these findings suggest that the epigenetic modulation of PDL-1 expression is associated with a profound modification of the tumor microenvironment (TME), favoring the antitumor immune response. This was confirmed by a stronger effect of anti-PDL-1 IC therapy in mice treated with a combination of OXP and DAC compared to mice treated with single drugs [12]. Overall, it is clear that chemotherapy and epigenetic drugs can influence ICI therapy.

## 4. Chemotherapy and ICI Therapy to Trigger the Immunogenic Cell Death Effect

CRC with microsatellite instability (MSI) can elicit a good immune response, at variance with microsatellite stable (MSS) CRC [13,14]. Indeed, MSI generates a large array of tumor-associated neoantigens, leading to the generation of specific T lymphocyte immune responses. It has been reported that high tumor mutational load, the amount of CD8+ TIL mainly at the tumor–stroma margin, and high PDL-1 expression may affect the therapeutic efficacy of ICI [13,14]. Importantly, the response rate to PD-1 inhibitors could be near 0% in mismatch-proficient patients, due to the very low tumor mutational load [15]. The efficacy of ICI is strictly associated with the presence of activated T cells that can correctly recognize tumor cells expressing tumor-specific antigens, conditioning a less immunosuppressive TME [16].

Several approaches could be pursued to improve the efficacy of ICI therapy in attempts to overcome drug resistance. The enhancement of T cell and antigen-presenting cell (APC) recruitment and the improvement of T cell recognition within the tumor can be useful to improve the response to ICI therapy in CRC. Chemotherapy, either alone or associated with epigenetic drugs, can be an additional tool to increase the immunogenicity of the tumor. Indeed, this treatment can increase tumor-associated antigens and consequently the antitumor immune response. A similar effect would be elicited using oncolytic viruses, hyperthermia, or photodynamic therapy. Importantly, chemotherapy, radiotherapy, and physical treatments can lead to immunogenic cell death (ICD) [17]. During ICD, tumor antigens, danger-associated molecular patterns (DAMPs), high-mobility group box-1, adenosine triphosphate, type I IFN, and heat shock protein (HSP) are released into the TME. After engagement with their counter receptors on several leukocyte subpopulations, DAMPs induce an immune response and consequently immune cell memory [17]. 

## 5. Conclusions

It is evident that the combination of the various therapeutic schemes described above with ICI therapy is the next step, already ongoing, to improve the immune response and consequently ICI therapy effectiveness. This approach could also be implemented by the combination of ICIs directed at different receptors or their corresponding ligands, further increasing their responsiveness in resistant/refractory patients. The study of the role of IC on tumor cells is mandatory, as the target of ICI therapy is usually the lymphocyte, not the tumor cell. Theoretically, the engagement of IC molecules on the target would elicit effects comparable to those observed in leukocytes. Thus, the engagement of CTLA-4 or PD1 with specific ICI on tumor cells would inhibit the negative signal delivered through the receptor, triggering a pro-tumorigenic effect. Of course, this scenario is not well-defined and requires specific intracellular signaling studies. Several IC molecules and ligands can also be released as microvesicle/exosome components or as soluble molecules. These cell-free forms, in turn, could conceivably influence the net effect of ICI therapy, buffering the levels of active drugs [18]. Further studies on these topics will warrant a more positive clinical outcome than that achieved so far.
